# Supplementary material for: Cellular basis of accelerated whole-tooth regeneration
Source: eLife. 2026 Sep 24;15:RP110584. doi: 10.7554/eLife.110584 (PMC13609483; doi:10.7554/eLife.110584)
Supplement: Peer review history

# Editor report: eLife Assessment

**DOI:** 10.7554/eLife.110584.3.sa0

**Authors:** Casimira Valeria Chuquimez Ventura

**Affiliations:** Masaryk University Czech Republic

**Keywords:** Solid, Valuable

This study provides **valuable** insights into the cellular dynamics underlying accelerated tooth regeneration in a vertebrate model. Using single-nucleus RNA sequencing across multiple time points, the authors present a well-structured analysis of cell populations, trajectories, and intercellular signaling events associated with this process. The strength of evidence is **solid** but only partially supported, as the conclusions are primarily supported by computational inference, without experimental validation of key findings.

# Referee report: Reviewer #1 (Public review)

**DOI:** 10.7554/eLife.110584.3.sa1

Summary:

The authors used single-nucleus RNA sequencing (snRNA-seq) to investigate accelerated tooth replacement following tooth plucking in cichlid fish. They analyzed four stages of regeneration using elegant and well-designed approaches to characterize cellular trajectories and interactions within the dental epithelium and mesenchyme during the accelerated replacement process. Their analyses identified cell type-specific gene expression profiles and intercellular signaling interactions associated with whole-tooth regeneration.

Strengths:

This is a highly interesting and thoughtfully executed study that provides compelling and convincing insights into the mechanisms underlying accelerated tooth regeneration.

Comments on revised version.

I noted in my initial review that "the manuscript currently lacks experimental validation of the single-nucleus RNA-seq data." In response, the authors have added a statement indicating that their cell-type annotations and pathway interpretations are supported by extensive prior experimental work in the cichlid tooth model, including histology, in situ hybridization, immunohistochemistry, and pharmacological perturbation of major developmental pathways. They have also acknowledged this limitation in the Study Limitations and Future Directions section, stating that direct experimental validation of the single-nucleus RNA-seq findings will be the focus of future studies.

The authors have carefully addressed my comments, particularly the Major Points (2), (3), and (4), as well as all of the Minor Points. I appreciate their efforts to further characterize the mesenchymal landscape surrounding the putative successional lamina and to provide additional evidence supporting the presence of a specialized stromal microenvironment associated with tooth regeneration. Overall, the revisions have substantially strengthened the manuscript.

# Referee report: Reviewer #2 (Public review)

**DOI:** 10.7554/eLife.110584.3.sa2

Summary:

Mubeen and colleagues study the cellular basis of tooth regeneration in cichlid fish. Using an elegant tooth plunking strategy followed by single nucleus RNA-sequencing, the authors were hoping to achieve an atlas of cellular and transcriptional changes that occur within and between cells during whole tooth replacement.

Strengths:

The major strengths of the methods and results are high novelty in the approach in a vertebrate with continuous tooth replacement, the temporal analysis of analyzing at plucking and three later time points, the thorough and sophisticated analysis of the snRNA-seq data including the inferring of trajectories and signaling events, and the robust signal of transcriptional differences induced by tooth plucking.

Weaknesses:

The major weaknesses of the methods and results are no validation of any of the inferred cell types, no functional tests of whether any of the changes in signaling pathways affect the plucking-induced tooth replacement process, and perhaps no clear take-away message for biologists not necessarily interested in tooth replacement.

Conclusions:

The authors achieved their aims of identifying the changes in gene expression and cellular composition that occur during whole tooth replacement accelerated by plucking. Overall, the results support their conclusions, although some slight semantic qualifiers should probably be added (e.g. referring to "cell types" as "putative cell types").

The work should have high impact in the field of tooth and organ regeneration, and the novel methodological paradigm established here of accelerating tooth replacement three-fold by plucking has great promise for future follow up studies to further study this process. The work also could have strong impact by the computational methods used here to infer trajectories and signaling interactions. Specific pathways, genes, and cell types could be tested in other fish such as zebrafish to test function during tooth replacement.

The work is unique and interdisciplinary and also has significance by establishing that robust phenotypically plastic accelerations in regeneration rates occur upon tooth removal. There are very few studies like this one that combine genetic x environmental studies of regeneration. The result that three different species of cichlid fish that normally have very different tooth patterns all accelerate tooth replacement threefold upon tooth plucking also has significance in revealing a highly conserved plucking response.

# Author comment: Author response

**DOI:** 10.7554/eLife.110584.3.sa3

**Authors:** Talha Mubeen, Haowen He, George Gruenhagen, Anoushka Satoskar, Jeffrey Todd Streelman

**Affiliations:** Georgia Institute of Technology Atlanta United States; Georgia Institute of Technology Atlanta United States; University of California, San Francisco San Francisco United States; Georgia Institute of Technology Atlanta United States; Georgia Institute of Technology Atlanta United States

The following is the authors’ response to the original reviews.

Many thanks to the three reviewers and the editors for their thoughtful comments and careful evaluation of our manuscript. These are fair, consistent and largely expected comments. On behalf of my co-authors, we provide this response to the public reviews to summarize the main issues raised and the corresponding revisions we have made in the revised manuscript.

(1) The main consistent comment from all three referees was that our single-nucleus RNA-seq data should be further validated. The reviewers differ in the detail of exactly what they think should be validated, but collectively these comments referred to validation of: (1) the identified cell types, (2) pathways inferred from trajectory analysis, (3) differentially expressed genes between plucked and control conditions across the four sampled time points, and/or (4) inferred ligand–receptor pairs from the cell–cell communication analysis.

We believe that we are on strong footing for some of these points because of extensive work we’ve done in the past in the cichlid fish model.

In the references cited in the manuscript and highlighted below (References 1, 10, 11, 29, 30, 31), we tally 29 figures with 273 individual figure panels presenting histology, in situ hybridization, and immunohistochemistry featuring genes expressed in cichlid (replacement) teeth. Most of these genes are markers of dental competency and/or indicative of regenerative potential.

In addition, in multiple of these papers, we use pharmacology to manipulate the role of key pathways (Hh, BMP, Wnt, Notch) in cichlid tooth development and replacement. Validation of cell types in the present study therefore draws on these published data in cichlids (and other vertebrates), as well as on an unbiased comparative approach, SAMap, which identifies homology between cichlid and mouse dental cell types based on shared gene expression.

In short, experiments to validate cell types and pathways active in cichlid teeth have been published and are referenced herein. We recognized, however, that these references (some of which include Gareth Fraser as an author, when he was a postdoc in my group; for Reviewer 2) were cited primarily in the Introduction, rather than in the Rationale/Methods or Results sections. We have therefore clarified these connections in the revised manuscript (line 173-74).

We have not validated nor analyzed functionally the ligand-receptor pairs we inferred from cell-cell communication analysis. This work is beyond the scope of the current paper, and we now state more clearly that these inferences represent hypotheses to be tested in future studies, although many of these ligand–receptor pairs have been noted in other tooth-related publications cited in the manuscript.

(2) The biggest weakness of our manuscript, noted by referees, is that we do not provide serial histology to accompany our snRNA-seq time course after plucking. We previously described this as a limitation in the “Study limitations and future direction” section of the Discussion, but we have now strengthened this discussion. In particular, we more explicitly acknowledge that we do not directly document the histological progression of tissue responses across the plucking time course or the degree of tissue damage caused by the plucking paradigm at each sampled time point.

In the “study limitations” section, we note both issues 1 and 2 and suggest that a spatial transcriptomics experiment across the timespan of plucking<>recovery would address simultaneously the desire to understand cellular context of plucking and cellular/spatial differences in plucked vs control cell-type gene expression.

(3) Reviewers also asked about the presence and interpretation of stromal cells in our snRNA-seq data. In response, we re-examined the mesenchymal compartment and added additional analyses to better characterize stromal/mesenchymal populations and their inferred trajectories in the revised manuscript. This includes a revised Figure 4, revised text around Figure 4 and revised Supplementary Figures.

(4) Multiple (minor) suggestions for clarification in text and figures have been adopted throughout the revised manuscript, figure legends, and supplemental materials.

Overall, we do not anticipate that further reviewer engagement will be necessary, and we believe that editorial review of the revised manuscript should be sufficient.

References cited in the manuscript, highlighted here:

(1) Fraser, G. J. et al. An Ancient Gene Network Is Co-opted for Teeth on Old and New Jaws. PLoS Biol. 7, e1000031 (2009).

(10) Fraser, G. J., Bloomquist, R. F. & Streelman, J. T. Common developmental pathways link tooth shape to regeneration. Dev. Biol. 377, 399–414 (2013).

(11) Bloomquist, R. F. et al. Developmental plasticity of epithelial stem cells in tooth and taste bud renewal. Proc. Natl. Acad. Sci. 116, 17858–17866 (2019).

(29) Streelman, J. T., Webb, J. F., Albertson, R. C. & Kocher, T. D. The cusp of evolution and development: a model of cichlid tooth shape diversity. Evol. Dev. 5, 600–608 (2003).

(30) Fraser, G. J., Bloomquist, R. F. & Streelman, J. T. A periodic pattern generator for dental diversity. BMC Biol. 6, 32 (2008).

(31) Bloomquist, R. F. et al. Coevolutionary patterning of teeth and taste buds. Proc. Natl. Acad. Sci. 112, (2015).

**Public Reviews:**

**Reviewer #1 (Public review):**

Summary:

The authors used single-nucleus RNA sequencing (snRNA-seq) to investigate accelerated tooth replacement following tooth plucking in cichlid fish. They analyzed four stages of regeneration using elegant and well-designed approaches to characterize cellular trajectories and interactions within the dental epithelium and mesenchyme during the accelerated replacement process. Their analyses identified cell-type-specific gene expression profiles and intercellular signaling interactions associated with whole-tooth regeneration.

Strengths:

This is a highly interesting and thoughtfully executed study that provides compelling and convincing insights into the mechanisms underlying accelerated tooth regeneration.

Weaknesses:

The manuscript currently lacks experimental validation of the single-nucleus RNA-seq data.

We thank Reviewer #1 for the thoughtful and positive assessment of our study, including the recognition that our snRNA-seq time course provides insight into cellular trajectories, cell-type-specific gene expression, and inferred intercellular signaling during accelerated tooth replacement in cichlid fish. We also appreciate the reviewer’s central concern that the manuscript would be strengthened by additional experimental validation of the single-nucleus RNA-seq data.

As summarized above and discussed in more detail in our point-by-point responses below, we have clarified how the present cell-type annotations and pathway interpretations are supported by extensive prior experimental work in the cichlid tooth model, including histology, in situ hybridization, immunohistochemistry, and pharmacological perturbation of major developmental pathways. We have also added analyses demonstrating reproducibility across biological test subjects and consistency of representative differentially expressed genes between paired plucked and control samples. Finally, we have strengthened the Study Limitations section to more clearly state that future spatial transcriptomic, histological, and functional validation experiments will be important next steps.

**Reviewer #2 (Public review):**

Summary:

Mubeen and colleagues studied the cellular basis of tooth regeneration in cichlid fish. Using an elegant tooth plunking strategy followed by single-nucleus RNA-sequencing, the authors were hoping to achieve an atlas of cellular and transcriptional changes that occur within and between cells during whole tooth replacement.

Strengths:

The major strengths of the methods and results are high novelty in the approach in a vertebrate with continuous tooth replacement, the temporal analysis of analyzing at plucking and three later time points, the thorough and sophisticated analysis of the snRNA-seq data, including the inference of trajectories and signaling events, and the robust signal of transcriptional differences induced by tooth plucking.

Weaknesses:

The major weaknesses of the methods and results are no validation of any of the inferred cell types, no functional tests of whether any of the changes in signaling pathways affect the plucking-induced tooth replacement process, and perhaps no clear takeaway message for biologists not necessarily interested in tooth replacement.

Conclusion:

The authors achieved their aims of identifying the changes in gene expression and cellular composition that occur during whole tooth replacement accelerated by plucking. Overall, the results support their conclusions, although some slight semantic qualifiers should probably be added (e.g., referring to "cell types" as "putative cell types").

The work should have a high impact in the field of tooth and organ regeneration, and the novel methodological paradigm established here of accelerating tooth replacement three-fold by plucking has great promise for future follow-up studies to further study this process. The work could also have a strong impact through the computational methods used here to infer trajectories and signaling interactions. Specific pathways, genes, and cell types could be tested in other fish, such as zebrafish, to test function during tooth replacement.

The work is unique and interdisciplinary, and also has significance by establishing that robust phenotypically plastic accelerations in regeneration rates occur upon tooth removal. There are very few studies like this one that combine genetic and environmental studies of regeneration. The result that three different species of cichlid fish that normally have very different tooth patterns all accelerate tooth replacement threefold upon tooth plucking also has significance in revealing a highly conserved plucking response.

We thank Reviewer #2 for the careful and constructive evaluation of our manuscript and for highlighting the novelty of the cichlid tooth-plucking paradigm, the temporal design of the snRNA-seq experiment, and the computational analyses used to infer cellular trajectories and signaling interactions during accelerated tooth replacement. We also appreciate the reviewer’s comments regarding validation of inferred cell types and interpretation of signaling pathways.

In response, we have revised the manuscript to clarify that our cell-type annotations are supported by marker-gene expression, previously published cichlid tooth studies, and an unbiased comparative approach, SAMap, which relates cichlid and mouse dental cell types based on shared gene-expression structure. We have also clarified that inferred ligand-receptor interactions represent computational hypotheses rather than functionally validated mechanisms. In addition, we revised Figure 6 and Figure 7A to improve the readability and interpretation of inferred signaling results, and we edited the relevant text and figure legends to make these results easier to follow. These points are addressed in greater detail in the point-by-point responses below.

**Reviewer #3 (Public review):**

Summary:

This is an interesting paper. The process of tooth exfoliation and replacement in vertebrates remains an intriguing and fascinating subject of inquiry. As the scientists noted, there are no mammalian models that can be used to examine signaling pathways in real time.

Strengths:

This work integrates in vivo and high-resolution transcriptomics. The study confirms previous findings and emphasizes the need for additional research into the processes that drive the restoration of missing teeth for future therapeutic uses.

Weaknesses:

I disagree with the use of the phrase "plucking". Instead, the authors use tooth extraction or tooth removal, which is clinically more correct for the procedure they are doing.

The inspiration for our ‘plucking’ experiment is work done in the hair follicle model (lines 73-74). Because cichlid teeth are so numerous, are very small, and lack dental roots, this is an accurate description of the procedure. We opt to retain the phrasing.

The title is rather broad and appears to be more appropriate for a review than an original research work. I would advise specifying the species under research and/or the sort of damage model used in the transcriptome analysis.

We opt to retain the title.

It's uncertain whether the findings are exclusively based on regeneration. The presence of tooth remnants, as well as unintended harm to surrounding tissues, may have triggered repair mechanisms, thereby biasing the current data. How did the authors handle this issue? The oral cavity was under severe manipulation, increasing the inflammatory stimuli, a situation that does not take place in physiological exfoliation.

In the revised manuscript, we have more clearly acknowledged that our plucking paradigm may induce tissue damage and repair-associated responses in addition to accelerated tooth replacement. We have strengthened the Study Limitations section to state that we do not directly document the histological progression of tissue responses across the time course or the degree of damage caused by plucking at each sampled time point. One caveat, however, is that bone remodeling and immune response is likely triggered on the ‘control’ side of the jaw also, just not to the same degree as after plucking.

The authors indicated the use of microCT analysis; however, no such information appears in the main text. In fact, this manuscript lacks anatomical information. It is required to conduct histological examinations of the regenerated teeth at various time points.

microCT data were included as a Supplemental Figure to demonstrate the dental formulae of our chosen species; but we did not characterize post-plucking recovery using this technique (see above summary and below point-by-point comments).

Although the current findings confirm previously found and verified signaling pathways, the absence of functional data lends uniqueness to this work.

In the revised manuscript, we also clarify that, while our transcriptomic analyses identify candidate cell states, pathways, and signaling interactions associated with accelerated replacement, the functional roles of these inferred pathways remain to be verified in future studies.

**Recommendations for the authors:**

**Reviewer #1 (Recommendations for the authors):**

Major Points:

(1) Figure 1 should include representative H&E staining images comparing the left control side and the regenerated side at 7 days post-plucking. This would provide important histological context for the regeneration process and help readers better interpret the molecular findings.

This would indeed be valuable information, but we did not carry out histology of paired control vs plucked jaws to accompany our pulse-chase and dissections for single-nucleus isolation. This comment is similar to that below about validation of what is happening on plucked vs. control jaw halves and is the first limitation we discuss in the “study limitations and future directions” section (from line 538).

(2) Each tooth position consists of a functional tooth, a replacement tooth, and the dental (successional) lamina. On the control side, the successional lamina contains teeth at different developmental stages, analogous to the mammalian bud, cap, and bell stages. Can the snRNA-seq analysis distinguish among tooth families at different developmental stages, as well as the individual components within a single tooth unit? Clarification of this point would enhance the developmental interpretation of the dataset.

No, our approach does not distinguish among teeth at different stages, nor among teeth in even vs odd positions that tend to be synchronized in replacement cycles. Theoretically, one could do this by dissecting individual teeth and pooling by tooth stage, but we did not.

(3) Identifying successional lamina cells is critical, and the authors report putative SL cells within the VEE cluster. However, the stromal cells surrounding the successional lamina are also known to play important roles in tooth regeneration. Can the authors further annotate and characterize stromal cell populations in the snRNA-seq dataset? Additional analysis of these supporting cells would strengthen the conclusions regarding epithelial-mesenchymal interactions.

We thank the reviewer for this insightful suggestion. In response, we further characterized mesenchymal subpopulations and included these new analyses in the revised manuscript (updated Figure 4 and Supplemental Figure 8). Specifically, pseudotime and CellRank analyses identified a mesenchymal subpopulation enriched for *Twist1*, *Dnmt1*, and *Runx2*, which we interpret as putative dental ectomesenchyme (DEM) based on the established roles of these genes in odontogenic mesenchymal development and differentiation, as well as their reported expression in mouse and human tooth single-cell transcriptomic studies. Notably, this putative dental ectomesenchymal population resides within the broader dental follicle compartment identified in our dataset (Figure 4A-C).

To further assess supporting stromal populations, we examined the expression of established stromal marker genes, including *Lum*, *Col6a3*, *Aspn*, and *Vegfc*. These markers were broadly restricted to mesenchymal populations and showed strong enrichment overlapping the newly identified putative dental ectomesenchymal region (Supplemental Figure 8B). Consistent with these observations, differential expression analysis identified additional DEM-enriched genes that substantially overlap canonical stromal markers, including extracellular matrix-associated genes, supporting a close transcriptional relationship between the putative dental ectomesenchyme and the surrounding stromal mesenchymal compartment (Supplemental Figure 8C). Together, these findings refine the mesenchymal landscape surrounding the putative successional lamina and support the presence of a specialized stromal microenvironment associated with tooth regeneration.

Consistent with this interpretation, our CellChat analysis identified significantly increased interactions between the dental ectomesenchyme and cycling ameloblast populations on the plucked side at Day 0 (Supplemental Figure 8D). These interactions were enriched for signaling pathways including SEMA4, EPHB, SLIT and SPP1, all of which have established roles in tissue remodeling, extracellular matrix organization, and regenerative processes. (Supplemental Figure 8E). Because Day 0 contained sufficient biological replicates and cell numbers for robust statistical comparison, we focused our interaction analyses on this time point. Collectively, these additional analyses provide a more comprehensive characterization of the stromal compartment and further support the conclusion that a specialized dental ectomesenchymal population actively participates in epithelial-mesenchymal communication during the earliest stages of tooth regeneration. So, in total, Figure 4 was revised, the text on lines 281-309 was revised, and Supplemental Figure 8 was added.

(4) The manuscript currently lacks experimental validation of the single-nucleus RNA-seq data. The authors should validate the expression of major signature genes using RNAscope or immunostaining, ideally comparing regenerated samples with the left-side control. Such validation would significantly enhance the robustness of the conclusions.

We did not validate up- or down-regulation of differentially expressed genes in intact tissue, owing in part to (1) the complexity of this experiment, (2) the fact that the majority of DEGs, or ‘major signature genes’ have been observed to be expressed in dentitions generally, and often by us in previous work on cichlid teeth, and the fact that (3) independent biological replicates were strongly consistent in the direction of effects (see below). In the “study limitations” section, we note this issue and suggest that a spatial transcriptomics experiment across the timespan of plucking<>recovery would address simultaneously the desire to understand cellular context of plucking and cellular/spatial differences in plucked vs control cell-type gene expression.

Minor Points

(1) In Figure 1, the color scheme used in the schematic drawing (Figure 1A) should match the corresponding structures shown in Figure 1B to improve clarity and consistency.

We appreciate the reviewer’s thoughtful suggestion regarding the color consistency between the schematic (Figure 1A) and the fluorescence images (Figure 1B). However, the color scheme in the schematic (Figure 1A) was intentionally selected to maximize accessibility, particularly for readers with color vision deficiencies, and therefore differs from the magenta and green fluorescence channels used in Figure 1B. In the fluorescence images, the magenta and green colors reflect the native display colors used for the Alizarin Red and Calcein labeling channels in the pulse-chase experiment. Directly matching the schematic colors to the fluorescence images could reduce the visual contrast between key anatomical structures and compromise accessibility for some readers. We have therefore retained the current color scheme in Figure 1A while ensuring that the corresponding structures are clearly identified through consistent labels and annotations across both panels.

(2) The abbreviation for successional lamina (SL) should be defined upon first use in the Introduction.

We thank the reviewer for catching this omission. We have now defined the abbreviation “successional lamina (SL)” upon its first appearance in the Introduction.

(3) Regarding biological replicates, the authors should provide data demonstrating the consistency and reproducibility across replicated samples.

We thank the reviewer for this suggestion. To demonstrate the consistency and reproducibility across biological test subjects, we have added analyses summarizing sequencing quality metrics, test subject contributions, integrated clustering, and representative differential gene expression across individual samples (see Figure S4, panels C, D & E and Author response image 1). Panel A shows that nuclei from different biological test subjects are well integrated across clusters rather than segregating by sample origin. Finally, Panel B presents representative differentially expressed genes from multiple cell populations, demonstrating consistent expression differences between paired plucked and control samples across biological test subjects.

**Author response image 1.** Single-nucleus RNA-seq data demonstrate consistency across biological replicates in dental cell-state representation and differential gene expression. (A) UMAP embedding of dental nuclei. Each point represents a single nucleus, colored by test subject. (B) Representative differentially expressed genes show consistent expression differences between plucked and control samples across biological replicates. Paired boxplots of average gene expression for representative differentially expressed genes from multiple cell populations at Days 0, 1, 3, and 7. Each point represents one biological replicate (test subject), with paired plucked and control samples connected by dashed lines. The y-axis shows average gene expression, and the x-axis indicates the experimental condition. These representative examples illustrate the consistent direction of differential expression across biological replicates, supporting the reproducibility of the single-nucleus RNA-seq dataset.

**Reviewer #2 (Recommendations for the authors):**

(1) Figure 1: Can the panels to the right of panel B be labeled? It's not clear what these six images are showing, so giving them letters and explaining briefly in the legend what the point of each panel is would clarify. "Right, example of individually classified teeth" - can the authors elaborate on what each tooth is an example of (i.e., how each tooth shown was classified"?) For clarity, the graphs in panels C and D should have the y-axes labeled

We thank the reviewer for this helpful suggestion. In response, we revised the Figure 1B legend to clarify the classification criteria used for dye incorporation analyses and to better describe the representative fluorescence images. Specifically, teeth positive for both Alizarin and Calcein were classified as pre-existing old teeth, whereas teeth positive only for Calcein were classified as newly formed teeth. We additionally clarified that the images to the right of panel B show representative individually classified teeth, with the top row representing pre-existing old teeth and the bottom row representing newly formed teeth. We also added y-axis labels to panels C and D to improve figure clarity and readability.

(2) Figure 2 legend: should "the cell type" instead be "the putative cell type"? Without validation for all cell types, it seems adding some sort of qualifier is in order here. Can the authors comment further on examples of validation from other studies? For example, Gareth Fraser has published numerous studies that show Pitx2 expression marking dental epithelium in different fish, yet none of these older papers are cited.

Identification and validation of cell types make use of multiple published datasets in cichlids (for markers matched to mouse), as well as an unbiased computational approach (SAMap) that draws homology between cichlid and mouse dental cell types, based on shared global patterns of gene expression. There is perhaps a philosophical debate to be had about the validity of ‘cell types,’ generally, but our data are validated using two methods. We edited the text in lines 167-177 to clarify, including citing references to our own work (these studies include Gareth Fraser as an author, when he was a postdoc with Streelman).

(3) Figure 6 is extremely complicated. Can any portions of rows or columns in these tables be highlighted in the figure to help the reader follow the proposed signaling interactions highlighted in the text?

We thank the reviewer for this helpful suggestion. To improve the readability of Figure 6 and better guide readers through the dynamic signaling patterns described in the text, we revised the figure by visually highlighting the key sender-receiver interaction regions discussed in the Results. Specifically, we annotated the interactions involving mesenchymal subpopulations and alveolar bone (OST) signaling toward CYC-AMB at Days 0 and 7, mesenchymal signaling toward NK/T cells at Day 1, and epithelial cross-talk centred around ES-2 at Day 3. These visual annotations allow readers to more readily identify the signaling interactions highlighted in the text and relate them to the corresponding regions of the interaction heatmaps.

(4) In Figure 7A, what does the black font indicate (if grey is up in control and red is up in plucked)? I'd guess not up in either, which then makes it unclear whether the sets in black are different or why they are being presented.

We thank the reviewer for pointing out this ambiguity. In Figure 7A, blue and red labels indicate signaling pathways identified by CellChat as condition-specific, with blue representing pathways detected only in the control condition and red representing pathways detected only in the plucked condition. In contrast, pathways shown in black represent signaling pathways detected in both conditions but exhibiting significant differences in inferred communication probability between conditions. Thus, the black labels denote shared signaling pathways whose activity differs significantly between control and plucked samples, rather than pathways unique to either condition. We have revised the figure legend to clarify this distinction and improve interpretability.

**Reviewer #3 (Recommendations for the authors):**

(1) I encourage the authors to offer information on the histological differences between teeth during physiological and accelerated replacement. I'm curious if the eruption's accelerated rate has any effect on the mineralization of those teeth.

We did not examine the histology of individual teeth, and so can’t comment on differences in mineralization.

(2) The findings section contains multiple sentences that should be moved under material and techniques.

We expect the reviewer is referring to paragraph lines 104-114, which was a tricky paragraph to place in the manuscript. In the end, we believe it represents important context necessary to interpret findings (which could be missed if moved to ‘methods’) and so we’ve chosen to keep this paragraph in its place.

(3) It would be useful to include a table showing sample distribution by experimental design.

We thank the reviewer for this suggestion. Sample distributions across experimental conditions, time points, biological test subjects, and identified cell populations are already provided in Supplementary Table 1. To improve clarity and accessibility, we have revised the table legend to more explicitly describe the experimental design and sample annotations represented in the table.

(4) The writers did a nice job with the graphics in Figure 8; however, the schematics in Figure C are difficult to follow and are not adequately discussed anywhere. Please note that this text may be of great interest to the dentistry community, including clinicians, and that a clear and succinct explanation of the schemes at the end would be quite beneficial.

We thank the reviewer for this helpful suggestion. We have revised the Figure 8 legend to more clearly explain Panel C as a summary schematic of inferred cell–cell communication events associated with accelerated tooth replacement after plucking. The updated legend clarifies that the pathway labels in Figure 8C summarize results directly from Figure 7A: red pathway labels indicate plucked-only signaling events, corresponding to pathways shown as full red bars in Figure 7A, while black pathway labels indicate signaling interactions detected in both plucked and control conditions but showing significant differences in interaction probability between conditions. Panel C also includes a cell-type legend at the bottom to identify the relevant cell populations.
